# The mystery of claustral neural circuits and recent updates on its role in neurodegenerative pathology

**DOI:** 10.1186/s12993-021-00181-1

**Published:** 2021-07-07

**Authors:** Vladimir N. Nikolenko, Negoriya A. Rizaeva, Narasimha M. Beeraka, Marine V. Oganesyan, Valentina A. Kudryashova, Alexandra A. Dubovets, Irina D. Borminskaya, Kirill V. Bulygin, Mikhail Y. Sinelnikov, Gjumrakch Aliev

**Affiliations:** 1 Sechenov University, 11/10 Mokhovaya St, Moscow, 125009 Russia; 2 Center of Excellence in Molecular Biology and Regenerative Medicine (CEMR), Department of Biochemistry, JSS Medical College, JSS Academy of Higher Education and Research (JSS AHER), Mysuru, Karnataka India; 3 Research Institute of Human Morphology, Moscow, 117418 Russia; 4 Moscow State University, Vrorbyebi Gori, Moscow, Russian Federation

**Keywords:** Claustrum, Ontogenesis, Information processing, Neural networks, Neurodegenerative disorders

## Abstract

**Introduction:**

The claustrum is a structure involved in formation of several cortical and subcortical neural microcircuits which may be involved in such functions as conscious sensations and rewarding behavior. The claustrum is regarded as a multi-modal information processing network. Pathology of the claustrum is seen in certain neurological disorders. To date, there are not enough comprehensive studies that contain accurate information regarding involvement of the claustrum in development of neurological disorders.

**Objective:**

Our review aims to provide an update on claustrum anatomy, ontogenesis, cytoarchitecture, neural networks and their functional relation to the incidence of neurological diseases.

**Materials and methods:**

A literature review was conducted using the Google Scholar, PubMed, NCBI MedLine, and eLibrary databases.

**Results:**

Despite new methods that have made it possible to study the claustrum at the molecular, genetic and epigenetic levels, its functions and connectivity are still poorly understood. The anatomical location, relatively uniform cytoarchitecture, and vast network of connections suggest a divergent role of the claustrum in integration and processing of input information and formation of coherent perceptions. Several studies have shown changes in the appearance, structure and volume of the claustrum in neurodegenerative diseases, such as Parkinson’s disease (PD), Alzheimer’s disease (AD), autism, schizophrenia, and depressive disorders. Taking into account the structure, ontogenesis, and functions of the claustrum, this literature review offers insight into understanding the crucial role of this structure in brain function and behavior.

## Introduction

The claustrum is one of the least understood anatomical structures within the cerebral hemispheres of the brain. It is located between the cortex and putamen, which are separated from the claustrum by the extreme and external capsules [1]. The claustrum consists of a specialized network of cells with connections to cortical and subcortical regions [1]. Several questions regarding claustrum network functioning and involvement in neurological or neurodegenerative diseases remain unanswered. What functions do claustrum neurons mediate when relaying cortical signals? Why do some diseases affect the structure and volume of the claustrum? While specific answers to these questions are still unavailable, we combine and analyze published findings in search for solutions. Studies have shown that claustrum dysfunction is seen in several neurological diseases, but specific changes of the claustrum network and it’s connections remain undiscovered [2–5].

The complex functional nature of the claustrum is reflected through the variable nomenclature used for its description [6]. It is generally believed that the first illustration of the claustrum was made in 1786 by the French anatomist Felix Vicq d’Azyr who referred to it as “the taeniaformis nucleus” [1, 5, 6]. According to other sources, the claustrum was identified in humans in 1672 by Thomas Willis [5]. The first description of the claustrum was made in the early 19th century, termed “the vormauer” by Karl Friedrich Burdach [5]. This was the earliest report describing the structure and functions of the claustrum. Later, Burdach renamed the described structure as “the claustrum” [7]. Theodor Meinert, was the first who suggested the possible role of the claustrum in information processing [4, 8].

The claustrum network was further studied by Golgi, Nissl, Weigert, Marchi, Ramón y Cajal, and Brodman who studied the structure and anatomical relations of the claustrum. Current technological advances enable application of new methods to study its structure and functions [7]. As such, the specific role of the claustrum in neural activity regulation, complex behavioral patterns, emergence of consciousness and neurodegenerative diseases is being actively explored [9–11].

## Materials and methods

We performed a systematic review of literature regarding the ontogenesis, phylogeny, anatomy, pathology, neural connectivity association with neurodegenerative diseases of the claustrum, using the Google Scholar, Elsevier, NCBI MedLine, and eLibrary databases. The following key words were used: “claustrum”, “neural network”, “neural circuit”, “neuroanatomy”, “anatomy”, “pathology”, “neurodegenerative”, “degeneration”. We studied the references and conducted a citation search. The PICO model formed the basis of the search strategy. Foreign language material was included in this study. Two co-authors independently selected, evaluated, and extracted data.

## Results

A total of 176 articles were primarily selected for review. Exclusion of articles published prior to the year 2000, with the exception of articles of significant historical value, provided us with a total of 71 articles of potential significance for review. After a reference search, another seven articles were included in the final review. As a result, a total of 78 articles were included in the following review. In order to better portray our results, we categorized the findings into six subsections.

### Ontogenesis and phylogenetics of the claustrum

From the phylogenetic viewpoint, the claustrum first appeared in the form of a distinguishable nucleus in marsupials and primitive primates [12], though the claustrum has been also identified in reptiles (as a sleep-regulatory unit) [36]. Brodmann, Ariëns Kappers, Sonntag and Woollard, Rose et al. believed that the claustrum was a derivative of the insular cortex. This assumption was supported by morphogenetic similarity of these structures [12, 13]. But this was disputed by Landau, Faul and Holmgren, who stated that the claustrum is related to the corpus striatum in origin. This hypothesis is supported by reports of abnormal cerebral cortex development with a completely absent insular cortex in one of the hemispheres, while the claustrum remained intact. Additionally, it has been reported that the claustrum develops prior to the insular cortex. These findings give credibility to statements supporting the subcortical origin of the claustrum [12].

Furthermore, the claustrum is not considered to be part of the cortical plate or corpus striatum. It is rather regarded as an intermediary between these structures. Several valid arguments in favor of this theory have been provided, stating that the claustrum developed as a result of neuroblast accumulation from the pallial matrix (which borders the corpus striatum) [12]. These neuroblasts do not reach the cortical plate during migration and are therefore situated at a certain distance between it and the corpus striatum. Other studies have also presented similar findings regarding the ontogenesis of the claustrum. As of today, the hybrid claustrum ontogenesis hypothesis is considered the most acceptable [12].

Analysis of specific genetic markers in the insular cortex and the claustrum show different genetic profiles, which underlines the separate ontogeny of these structures [61]. Interestingly, despite specific genetic profile differences between the insular cortex and the claustrum, these two areas have the most similarities in genetic expression, compared to the rest of the brain regions [61]. Nonetheless, existing data suggests that the claustrum is an independent ontological structure, characteristic of newer and advanced neural function development. Genetic studies have also presented data supporting the claim that the claustrum and amygdala are homologues of the sauropsid dorsal ventricular ridge, though epigenetic, hodological, morphological, and topographical data did not support this hypothesis [85]. The evaluated genetic links show valuable insight into the ontological formation of the mammalian claustrum.

### Gross anatomy of the claustrum

The claustrum is often referred to as the “wall of the brain”. It is a thin, curved sheet of neurons embedded in white matter, located in both brain hemispheres deep to the neocortex. The claustrum is situated between the insula and putamen, separated by the extreme and external capsules respectively [14]. The frontal part of the claustrum is directed towards the corpus amygdaloideum and is divided by fibrous bundles connected to the anterior commissure and ancyroid bundle.

**Fig. 1:**
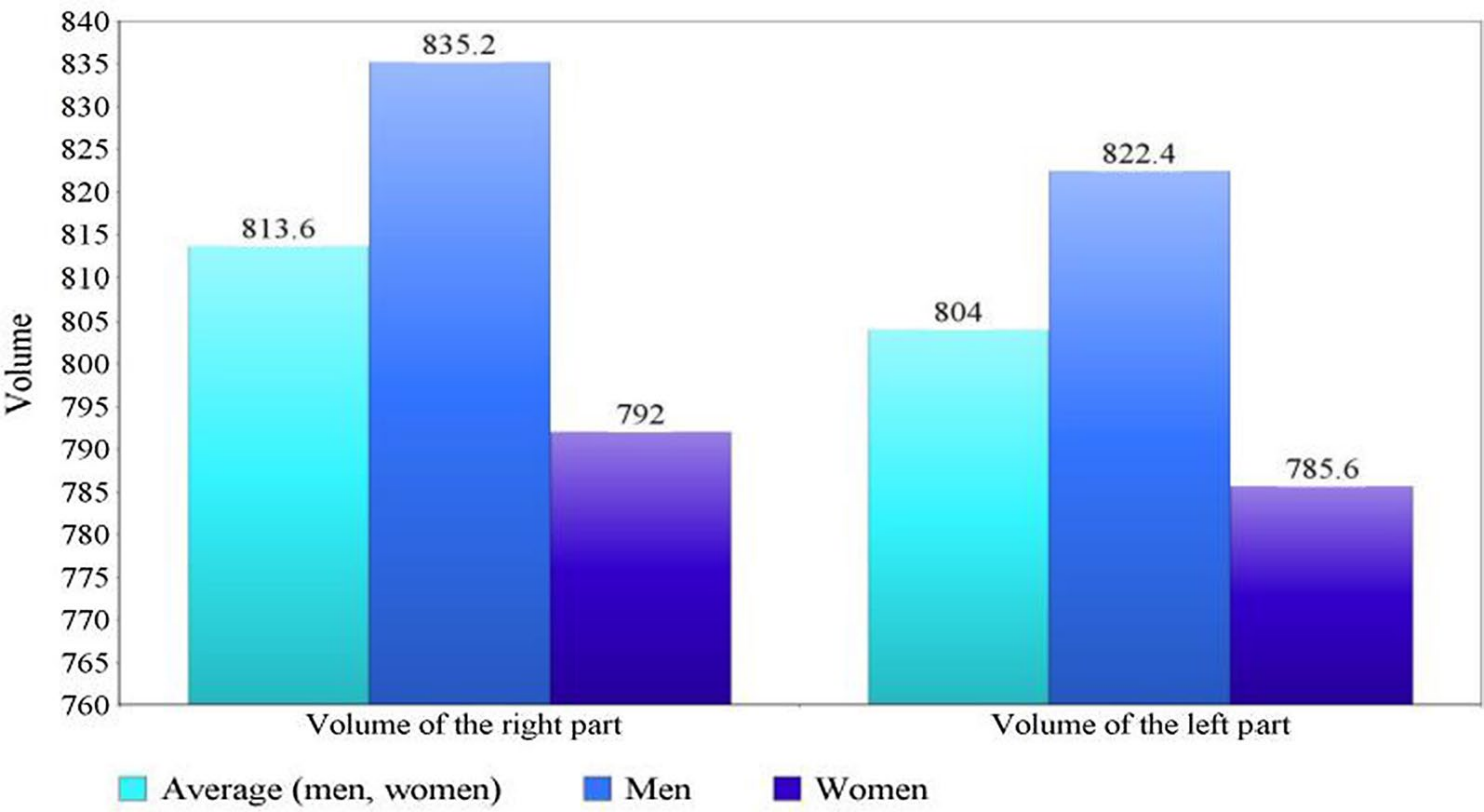
Claustrum volume discrepancies in the human brain

In humans, the claustrum occupies about 0.25% of the cerebral cortex volume [15]. Its thickness varies from a fraction of a millimeter to several millimeters [16]. Its dimensions are on average 38 mm rostrocaudally and 22 mm dorsoventrally [5, 15]. The inferior border of the claustrum is located at the level of the insular cortex and putamen [17]. The claustrum is asymmetrical, reflecting anisotropy between the hemispheres [17–19]. The volume of the claustrum ranges from 744 to 912 mm^3^. It has been reported that the average volume of the claustrum in men is greater than in women (Fig. 1). Claustrum volume asymmetry between hemispheres varies between 15 and 20%, with an average volume of 829 mm^3^ in the right hemisphere and 706 mm^3^ in the left [19].

Morphologically, the claustrum corresponds to the concavity of the insular cortex, and its inner curvature follows the convexity of the putamen. The claustrum is completely surrounded by white matter deep in the brain tissue, which complicates the study of its structure and connections. Exact anatomical boundaries of the claustrum still remain uncertain [18–21]. Blood supply to the claustrum is maintained through insular perforating vessels, deep and superficial branches of the medial cerebral artery [4, 16].

The claustrum is divided into the dorsal and ventral zones [22]. The dorsal (insular or compact) zone is located above the rhinal sulcus, medial to the insular cortex. The dorsal zone represents a continuous sheet of gray matter which expands inferiorly and superiorly [23, 24]. The ventral zone (referred to as the prepiriform, fragmented, amygdaloid, temporal claustrum or endopiriform nucleus [25]) is located medial to the piriform cortex and includes neural complexes underlying the rhinal sulcus [23, 24, 26]. The ventral zone is divided into the upper and lower sections. The upper ventral section is reported to be connected to the anterior pole of the dorsal zone, which extends to the base of the frontal lobe, and adjoins the prepiriform cortex. The lower ventral section connects to the posterior pole of the dorsal claustrum, which is directed towards the amygdaloid region of the cortex. Here the connection between the ventral claustrum and the amygdala is so close that it becomes difficult to differentiate these structures [24].

### Claustrum neural circuits

The presence of multiple classes of neuronal projections and interneurons are reported to exist within the mammalian claustrum [27, 28]. The anatomical proximity of the claustrum and its surrounding structures, underlines the difficulties in neuronal circuit differentiation. This may be due to mutual functional activity, but also due to the drawbacks of existing neural circuit evaluation methods (fMRI). Determining the connection between the registered signal and the activity of the actual neural substrate (claustrum, insula, or putamen) is difficult [8], since local stimulation may result in activation of nearby structures [29]. Small Region Confound Correction (SRCC) can be used to differentiate the claustrum neural circuits from nearby anatomical structures, due to high accuracy of the method [29]. The relationship between the structural and functional aspects of the claustrum still requires extensive research to develop methods that would eliminate existing limitations. Currently, such methods are being developed, allowing for potentially larger-scale more precise studies of the claustrum [30].

**Fig. 2:**
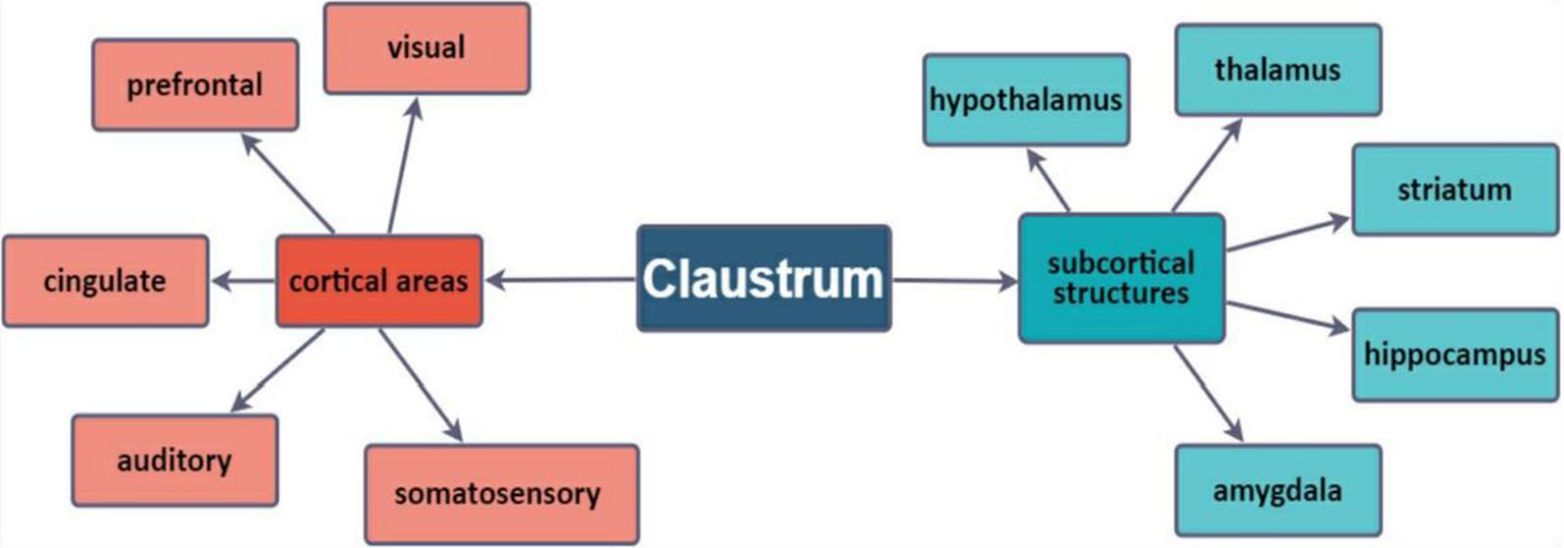
The schematic depiction of claustrum neuronal connections with cortical and subcortical structures

Several neuroanatomical studies showed that the claustrum has an extensive network of connections to subcortical regions, including the hippocampus, thalamus, putamen, and basal nuclei, as well as the temporal, occipital, and sensory lobes [18, 24, 31–33; Fig. 2). It remains to be determined if there are any cortical regions that are not connected with the claustrum [18, 19]. As such, the human claustrum is considered to be one of the most densely connected structures in the brain per unit volume [15, 31].

Existing tractography studies identified two main projection tracts: the dorsal and ventral. The dorsal tract is a group of fibers that connects the claustrum to sensory and motor regions of the cortex [24, 34]. The ventral tract connects the claustrum to the auditory and olfactory regions [5]. Other sources differentiate four fiber tracts connecting the claustrum to the cerebral cortex: the anterior, posterior, upper, and lateral tracts [18]. The anterior and posterior tracts connect to the prefrontal cortex and visual analyzer areas. The upper tract connects to sensorimotor regions and the lateral tract connects to the auditory cortex. The medial tract connects to the basal ganglia, specifically to the caudate nucleus, putamen, and globus pallidus [35]. Connections with the basal ganglia, however are debated: several input organization studies did not reveal claustrum connections with basal ganglia [87, 88]. Other animal studies also do not find connections between basal ganglia and claustrum. Additional studies have shown connections between the claustrum and the contralateral cerebral hemisphere [86], which include interhemispheric cortico-claustral fibers and inter-claustrum fibers. Tractography studies performed with diffusion tensor imaging, are not a precise way of assessing anatomical connectivity. As such, the true extent and connectivity of the claustrum network is yet to be shown.

Claustral neural circuits show high affinity to the frontal cortex, including the anterior cingulate gyrus, the prelimbic region and medial prefrontal cortex. The primary sensorimotor regions seem to be poorly connected to the claustrum [29]. Claustrum neural circuits functionally link the anterior cingulate gyrus to the visual and parietal cortex and these projections distinguish the inhibitory circuit of claustrum neurons involved in processing and transmitting of information [11]. The claustrum therefore connects with the frontal cortex, the anterior cingulate gyrus and the secondary visual cortex (B1/B2), the parietal associative cortex and coordinates spatial–temporal control of certain cortical areas [11].

The claustrum has been shown to be connected with the cingulate and prefrontal cortex to foster cognition influence [29]. Cognitive task performance is accompanied by the activation and deactivation of certain cortical regions to confer functional connectivity linked to claustrum circuits [29]. Therefore, the human claustrum is reported to play a significant role in cognition control, specific task control independent of sensory-motor processing. Existing studies show that cortico-claustral projections originate from areas of the frontal cortex including the orbitofrontal, cingulate, and secondary motor cortex. The somatosensory, auditory, and visual regions are minimally connected to the cortical-claustral inputs [20]. Cortical-claustral contralateral projections are reported to be much denser than cortical-claustral ipsilateral projections [30]. The claustrum also receives projections from several subcortical structures including the mediodorsal thalamus, basolateral amygdala, and hippocampus [20]. Evidence of intraclaustral connections exists through an extensive network along the Rostro-caudal axis [36].

Several markers were discovered to be specific to the claustrum, such as the g-protein Gamma-2 subunit (Gng2) and parvalbumin-immunoreactive (PV-IR) neuropil [6]. Gng2 expression and other immunohistochemical staining of parvalbumin opens up new possibilities to delineate underlying neuronal network dynamics of claustrum neural circuits [34].

Seventy-five percent of neurons projecting into the visual cortex were shown to be Type I neurons, and 25% were Type II neurons. Parietal oriented neural circuits included 43% Type I and 57% Type II neurons [10]. Among claustral neurons projecting into the anterior cingulate gyrus, 21% were Type I and 79% were Type II. The executive cortex (anterior cingulate gyrus) receives mostly projections from Type II neurons, while the visual area receives mostly projections from Type I neurons. This data shows claustrum potential in mediation of cortical functions [10].

### Neuronal characteristics within the claustrum

In contrast to the cortex, the claustrum does not exhibit a layered organization. There is relatively simple and uniform cytoarchitecture within a small number of cell types specific to the claustrum [37]. As such, the claustrum is mostly composed of glutamatergic neurons [38] evenly distributed across the claustrum. The diameter of a glutamatergic neuron body is 15–29 μm and the dendrites of these cells are covered with spinules and do not exhibit a preferred orientation [39]. Their axons protrude from the claustrum, mainly into the cerebral cortex. Glutamatergic neurons are excitatory neurons and include pyramidal, fusiform, or circular neurons. However, the functional subgroups of glutamatergic neurons specific to the claustrum have not been fully identified [40, 41]. GABAergic neurons are less widespread, and account for the remaining 15% of neurons within the claustrum [38]. The diameter of the GABAergic neural body is 10–15 μm [22]. Dendrites of these neurons appear smooth and axons do not extend beyond the body of the claustrum [38].

Golgi lipofuscin granular staining showed the presence of five types of neurons within the claustrum, consisting of a combination of spiny and aspiny nerve cells with divergent morphometric properties [42]. Type I cells are spiny neurons with small and widely distributed chromolipoid granules. Type II cells are large aspiny neurons and contain a large number of pigment granules. Type III cells are large aspiny neurons without pigment granules. Type IV cells small aspiny neurons with pigment deposits. Type V cells are small aspiny neurons without chromolipoid granules [42, 43].

Some claustrum neurons express Vglut2, which is a characteristic of subcortical cells [6]. Claustrum neurons can further be differentiated by parvalbumin expression into PV(−) and PV(+) interneurons. Furthermore certain electrophysiological properties including distribution of spike accommodation, maximum firing rate, capacitance, voltage of the resting membrane, and membrane resistance have shown to differ in different groups of claustrum neural cells [6]. Claustrum neurons show reactivity to cortical signals [11, 44]. Further evaluation of electrophysiological features of different claustrum neurons may help unravel the specifics of pathological changes within the claustrum associated with neurodegeneration [19, 45, 46].

A transcriptome-wide analysis of claustrum neural cell component provides the most specific differentiation of specific cellular profiles. As such, *NR4A2*, *NTNG2*, and *LXN* are thought to be more strongly expressed in the claustrum [61]. Furthermore, the specific expression patterns in the claustrum show enriched genes pertaining to severe intellectual disability, epileptic encephalopathy, intracellular transport, spine development, macroautophagy, smoking addiction. The *NR4A2* gene plays a role in dopamine regulation, and has been implicated in depression, drug addiction, Parkinson’s disease, schizophrenia and bipolar disorder [61].

### Claustrum function

**Fig. 3:**
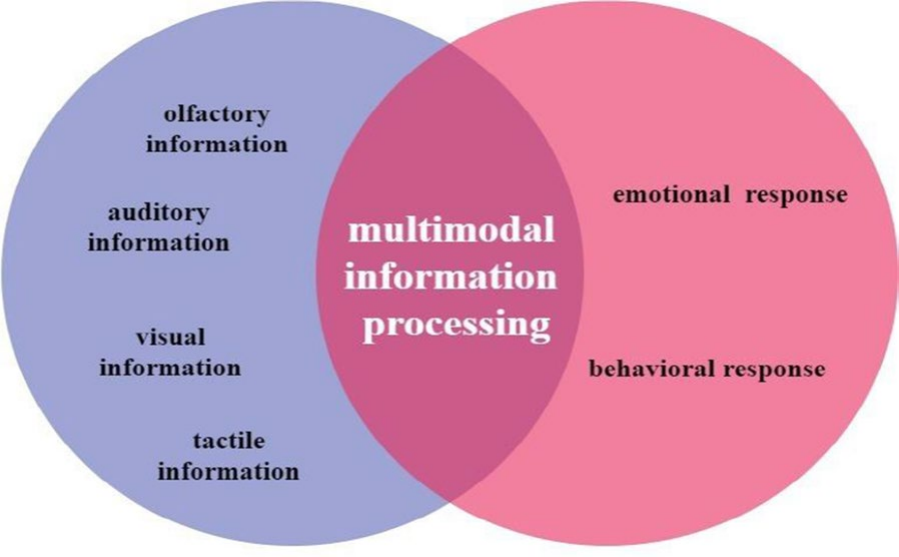
Multimodal information processing through the claustrum

Crick and Koch argued that the claustrum performs final integrative processing of input information, through convergence of several neural circuits connected to the cerebral cortex [47, 48]. The function of the claustrum is manifested when different sensory modalities are perceived altogether [49, 50]. It was found that the claustrum can combine visual, tactile, auditory, and emotional sensations (Fig. 3; 51, 52]. Furthermore, the claustrum plays a key role in neural mechanisms of consciousness [53–55].

Experimental studies show that inactivation of claustrum neurons typically undermined the efficiency to execute rewarding behavior due to increased susceptibility of such subjects to distracting factors [56]. Claustrum stimulation was found to cause a disruption in attentive behavior and a presumed loss of consciousness [57]. Existing reports suggest that the claustrum plays a significant role in mediating information processing in focus, prioritization and ignoring unnecessary sensory input [58, 59]. In monkeys, the claustrum has been shown to not have multi-sensory integration, with prevalence of unimodal responses [89]. As such, the claustrum plays a role in focusing of an individual’s attention on a current priority task or object [56, 60]. Fiber photometry studies in the auditory cortex suggested that activation of the claustrum suppresses auditory cortical responses to reduce sensory signals that do not have the current priority when performing a task. Thus, the claustrum modulates cortical information processing to isolate sensory information related to the task being performed [56].

The claustrum has also been shown to participate in memory storage, contiguity learning, suppression of natural urges, psychoses and recognition of fear [61–63]. The theory of the claustrum acting as a cortical-cortical relay center that supports attention is of significance [6]. Gng2/PV expression studies provided basis for understanding that the claustrum serves as an information filter for cortical projections and can act as a determination structure in recognizing the significance of incoming stimuli [6]. The claustrum has been shown to play a role in planned action control as it is involved in receiving information about planned movement from the frontal motor cortex and sensory cortexes [20]. Thus, the claustrum may play a role in integration of motor and sensory signals by relaying the processed signal back to the neocortex. It is emphasized that the inhibitory effect on cortical structures also plays a role in the regulation of claustrum functions and allows cortical circuits to encode stimuli according to specific behavioral patterns [20].

Studies have shown that D1r-expressing neurons that send projections to the frontal cortex are involved in determining stimulus significance [10]. In addition to neuromodulatory activity, the claustrum is involved in salience detection and attention. The projection of claustrum circuits into the frontal cortex leads to inhibition and forms a pattern of activity in frontal neurons, which contributes to the development of a connection between behavior and its context. This function might be mediated by dopamine receptors expressed in claustrum neurons [10]. Furthermore, a number of reports showed that gene expression in the claustrum is affected in cases of drug addiction, depression, severe mental retardation, seizures, and epilepsy [61, 64, 65].

Research indicates that there is a broad functional relationship between the claustrum and cortical areas that exercise cognitive control. Comparing the cognitive networks associated with task performance within claustrum related neural circuits, it was found that claustrum activation mainly occurs at the beginning of a task, when it is necessary to change the cognitive strategy or set a new goal [29]. The claustrum also plays a major role in controlling brain conditioning as it receives input from the midbrain and hindbrain. The claustrum is therefore ideally suited as a monitoring structure for sleep–wake cycle control and has been shown to be involved in sleep regulating neural circuits in reptiles and mammals [36, 90]. The claustrum may mediate integration of cortical and subcortical neural networks to produce conscious sensations [37]. Hodologic research studies depicted the salience detection and segregation of attention as another significant function mediated by claustrum [51, 56, 66]. Claustrum neural circuits play a role in mediation of sensory information, perpetual binding, and cognitive-related processes [67, 68].

Interestingly, several roles previously attributed to the claustrum have been shown to be specific to the insula, according to genetic profiling. Such functions include involvement in learning, mood disorders and dopamine signaling [61]. On the other hand, the claustrum has been shown to have unique gene upregulation profiles associated with depression, intellectual disability, spine development, epilepsy, encephalopathy, smoking addiction. These tendencies underline the role of the claustrum as a mediator of neural transmission, damage to which can lead to expression of depressive, cognitive and behavioral disorders.

### Role of the claustrum in neurodegenerative diseases

Through understanding of claustrum anatomical features, neural circuits, projections, histological aspects and function, the role of the claustrum in neurodegenerative diseases can be further defined. Anatomical asymmetry within the claustrum can be a sign of specific pathological behavioral patterns, including suppression of urges, pathological and deformed conditions [18].

The volume of the claustrum varies in different pathological conditions, such as schizophrenia, depressive disorders, Alzheimer’s disease (AD). Recent morphometric studies have found a bilateral decrease in the volume of the claustrum due to neuronal degeneration and is associated with both schizophrenia and severe depressive disorder [19]. It has been reported that the intensity of hallucinations in schizophrenia is inversely proportional to the volume of the claustrum [81]. In addition, measurements showed significantly smaller volumes of the claustrum in patients with paranoid schizophrenia comparative to those diagnosed with residual schizophrenia [19]. This data confirms that the claustrum may be affected structurally in schizophrenia and depressive disorders [46]. A decreased volume of the claustrum is also associated with the presence of delirium in patients with Alzheimer’s disease [19].

Claustrum changes in patients with Alzheimer’s disease is associated with progression of dementia and cognitive decline [50]. The pathophysiological changes during AD could be associated with pathology of neural circuits including the interhemispheric corticocortical and subcortical connections, partially associated with the claustrum. Damage to the integrating function of claustrum can affect memory, spatial orientation, transmission, and processing of information, and may cause delirium in AD patients [66, 69–71]. Additionally, the accumulation of amyloid plaques within the claustrum has been shown to play a direct role in the development of AD [4]. Amyloid deposits accumulating in the ventral zone of the claustrum can interrupt limbic connections [4].

Claustrum lesions are observed in almost all cases of Parkinson’s disease [72]. Cognitive complications of Parkinson’s disease thought to be mediated by claustrum disfunction are dementia, functional and behavioral impairment [73]. The occurrence of cognitive impairment in patients with Parkinson’s is associated with damage to white matter of the telencephalon [74], and the claustrum with its many connections, is believed to play a role in cognitive decline [29]. In patients with Parkinson’s disease, there is a decrease in claustral connectivity with cortical regions involved in visual-motor and auditory processing. There is a decrease in the density of connections with the parietal, upper temporal and postcentral cortical regions as well as the middle temporal gyrus, pars opercularis, pars triangularis, and pars orbitalis of the frontal gyrus [75]. Functional and anatomical disorders in claustrum are therefore associated with progressive dementia in Parkinson’s disease [75]. Atrophic lesions in the claustrum may be a manifestation of pathophysiological changes in patients with Parkinson’s disease [76, 77]. In Parkinson’s disease, the levels of dopamine and norepinephrine are significantly reduced within the claustrum [77]. Dopamine and norepinephrine deficiency in the claustrum may affect the mechanisms of information processing.

Claustrum disfunction has been associated with several neurological and clinical pathologies, including Wilson’s disease [78], epilepsy [79], Lewy body dementia [3, 80], schizophrenia [46], sleep disturbance, depressive symptoms, psychomotor retardation, anhedonia [72]. Some findings show that delusions and hallucinations in schizophrenia can be explained by the claustrum’s involvement in consciousness formation [81]. Structural damage to the claustrum may interrupt sensory processing and distort incoming information [82]. Studies have revealed that a significant delay in the growth of neurons accompanied by the deficiency of neuronal soma and general claustrum volume decrease is seen in autistic individuals [83]. The average claustrum volume in individuals with autism aged 4–8 years was 388 mm^3^, which is 22% less than that of a healthy cohort (494 mm^3^) [83]. As such, a significant decrease in the volume of the claustrum is seen in patients with autism [18, 83, 84].

The complexity and high connectivity of the claustrum neural circuits (claustrum network) is due to the central role of the claustrum in signal relaying and mediation of higher neural functions. The abundance of complex claustrum functions is associated with many possible disfunctions. The claustrum therefore plays an important role in neurodegenerative diseases, which are often associated with neural circuit pathologies. Further studies should implement specific circuit tracking to recognize claustrum-specific peculiarities.

## Conclusion

The claustrum remains a mysterious and poorly understood structure. The extensive neural circuit connectivity of the claustrum with cortical and subcortical regions suggests its crucial role in neural homeostasis. The claustrum plays an integrative role in processing of multisensory information to foster reward-related behavior. Detailed studies of the claustrum have proved difficult due to its complex shape and enclosed location. However, the latest research methods in neuroimaging, proteomics, and tractography helped obtain new information about the anatomical, physiological, and molecular features of the claustrum. Significant changes in the structure and volume of the claustrum are seen in a number of neurological conditions including: Wilson’s disease, epilepsy, AD, schizophrenia, Parkinson’s disease, Lewy body dementia, and autism. In this regard, further evaluation of claustrum-specific neural circuits and associated functions may lead to a better understanding of the causes and underlying pathologies of neurodegenerative diseases. Considering the claustrum as an integrating link in information processing, it can be assumed that damage through structural and functional lesions to claustrum networks could confer the disturbances in information processing. Finding targets for influence in these claustral neural circuits may help solve unresolved questions in neurodegenerative disease prevention and treatment. In conclusion, it is appropriate to assume that assessment of structural and functional changes within the claustrum may be beneficial to design novel therapeutic modalities and early detection methods of associated neurological diseases.

## Data Availability

All associated data is available from the corresponding author upon reasonable request.
